# Health and Disease: *Akkermansia muciniphila*, the Shining Star of the Gut Flora

**DOI:** 10.34133/research.0107

**Published:** 2023-04-03

**Authors:** Chen Xue, Ganglei Li, Xinyu Gu, Yuanshuai Su, Qiuxian Zheng, Xin Yuan, Zhengyi Bao, Juan Lu, Lanjuan Li

**Affiliations:** ^1^ State Key Laboratory for Diagnosis and Treatment of Infectious Diseases, National Clinical Research Center for Infectious Diseases, National Medical Center for Infectious Diseases, Collaborative Innovation Center for Diagnosis and Treatment of Infectious Diseases, The First Affiliated Hospital, Zhejiang University School of Medicine, Hangzhou, Zhejiang, China.; ^2^ Department of Neurosurgery, The First Affiliated Hospital, Zhejiang University School of Medicine, Hangzhou 310003, China.

## Abstract

*Akkermansia muciniphila* (*A. muciniphila*) has drawn much attention as an important gut microbe strain in recent years. *A. muciniphila* can influence the occurrence and development of diseases of the endocrine, nervous, digestive, musculoskeletal, and respiratory systems and other diseases. It can also improve immunotherapy for some cancers. *A. muciniphila* is expected to become a new probiotic in addition to *Lactobacillus* and *Bifidobacterium*. An increase in *A. muciniphila* abundance through direct or indirect *A. muciniphila* supplementation may inhibit or even reverse disease progression. However, some contrary findings are found in type 2 diabetes mellitus and neurodegenerative diseases, where increased *A. muciniphila* abundance may aggravate the diseases. To enable a more comprehensive understanding of the role of *A. muciniphila* in diseases, we summarize the relevant information on *A. muciniphila* in different systemic diseases and introduce regulators of *A. muciniphila* abundance to promote the clinical transformation of *A. muciniphila* research.

## Introduction

The gut is the main digestive organ of the human body and absorbs the most nutrients [1–3]. It is also the largest immune and detoxification organ [4]. The gut is also called the “gut brain” or “second brain” owing to its complex neural network of approximately 100 billion scattered nerve cells [5]. The function of the gut is closely related to human health. The gut harbors a vast microbial community, which plays key roles in gut function [6,7]. Gut microbes include bacteria, phages, viruses, protists, worms, and fungi. Bacteria are the main inhabitants of the gut and participate in physiological activities through intermediate metabolites or surface antigens [8,9]. The gut flora plays an important role in substance metabolism and immune defense and is necessary to maintain human health [10,11]. The gut flora communicates with the central nervous system through neural, immune, and endocrine pathways, affecting mood, behavior, and other brain functions [12]. The gut flora is associated with sleep, memory, anxiety, and depression [13–15]. Normally, the gut flora composition is relatively stable. Changes in the microbial abundance, composition, and levels of metabolites of gut flora lead to a variety of diseases, including cancer.

Recently, *Akkermansia muciniphila* has garnered attention for its potential role in inducing weight loss and reducing lipid levels and its age-delaying effects. *A. muciniphila* belongs to the mucin-degrading bacterial family and can generate energy by decomposing mucin secreted by the gut mucosa [16]. A reduced abundance of *A. muciniphila* can disrupt the gut barrier, leading to increased plasma endotoxin levels, abnormal inflammatory responses, and metabolic disorders. *A. muciniphila* has an important role in the occurrence and development of obesity, diabetes, inflammatory bowel disease, psychiatric diseases, aging, and other diseases. It could also improve the efficacy of immunotherapy for the treatment of some cancers. *A. muciniphila* has enormous potential to become a next-generation probiotic drug and therapeutic target. We aim to provide a systematic and comprehensive overview of the role of *A. muciniphila* in human health and disease.

## Shining Star: *A. muciniphila*

### The discovery and biological characteristics of *A. muciniphila*

In 2004, Derrien et al. identified a new mucus-degrading bacterium from human feces through anaerobic culture: *A. muciniphila*. It was named after Dr. Antoon Akkermans (1941 to 2006), a renowned microbial ecologist in Wageningen [17,18]. *A. muciniphila* is an oval, anaerobic Gram-negative bacterium. It has limited tolerance to oxygen, but preliminary laboratory operations can be carried out under aerobic conditions [19–21]. Amuc_1100 is a heat-stable outer membrane protein from *A. muciniphila*. It can withstand a high temperature of 70 °C [22]. In addition, *A. muciniphila* has a wide range of host adaptations and phenotypic diversity [23–27]. Geerlings et al. [24] isolated 10 *A. muciniphila* strains from the feces of 15 species of mammals. The genomes of these strains are highly similar, with a nucleotide sequence homology of 93.9% to 99.7%. A highly conserved mucin degradation gene ensures the mucin degradation ability of *A. muciniphila*. *A. muciniphila* is present in humans in breast milk and many digestive organs, such as the mouth, pancreas, gall bladder, small intestine, appendix, and colon. Dubourg et al. [28] also isolated *A. muciniphila* from blood culture samples.

*A. muciniphila* is originally obtained from the mother [29,30]. *A. muciniphila* makes use of oligosaccharides in breast milk by synthesizing key glycan-degrading enzymes, which contributes to the early colonization and survival of *A. muciniphila* in the gut [31,32]. The abundance of *A. muciniphila* in the gut increases rapidly during infancy and nearly reaches the abundance of *A. muciniphila* in adults by the age of 1 year [33,34]. Intestinal mucus is synthesized and secreted by goblet cells and forms an inner layer without bacteria and an outer layer containing symbiotic bacteria. *A. muciniphila* mainly colonizes the outer mucin layer of the gut and maintains the stability of the mucin layer by decomposing mucin [35]. The genome of *A. muciniphila* ATCC BAA-835T encodes more than 61 proteins related to mucin degradation, such as sialidases, β-galactosidases, and α-L-fucosidases [36,37]. β-Galactosidase (Amuc_0771, Amuc_0824, and Amuc_1666) can effectively degrade mucin-derived sugar chains by selecting different glycosidic bonds [37]. The highly active sulfatase of *A. muciniphila* promotes its gut colonization [38,39]. *A. muciniphila* can be grown on nonmucin sugar glucose medium, but its growth efficiency is significantly lower than when grown on mucin medium [17]. The 30 hydrolases involved in mucin degradation are significantly upregulated under mucin conditions [40]. N-acetylglucosamine transporters Amuc_0502 and Amuc_0516 are the most highly upregulated genes when *A. muciniphila* is grown on mucin medium. *A. muciniphila* produces a variety of metabolites such as oligosaccharides and short-chain fatty acids (SCFAs). Oligosaccharides can regulate metabolism by interacting with other bacteria. SCFAs, including acetic acid, propionic acid, and butyric acid, are important metabolites of *A. muciniphila* produced through anaerobic fermentation [41–43].

### The function of *A. muciniphila*

In addition to degrading mucin, *A. muciniphila* could also positively regulate mucin, thereby enhancing the barrier of the gut mucosa. Administration of *A. muciniphila* to mice increases the proliferation rate of intestinal stem cells, the number of goblet cells, and the number of Paneth cells [44,45]. *A. muciniphila* plays an important role in maintaining mucin homeostasis. In addition, mucin deficiency could result in the increased activation of glycolysis and other energy-generating metabolic pathways [46].

In vitro, *A. muciniphila* could increase enterocyte monolayer integrity and transepithelial electrical resistance to enhance the gut barrier [20]. Amuc_1100 activates Toll-like receptor 2 (TLR2) and its downstream NF-κB pathway [47]. Amuc_1100 can regulate metabolism and immunity and maintain the intestinal barrier [48]. Both butyric acid and propionic acid bind to G protein-coupled receptors to stimulate the secretion of intestinal peptides that regulate diet and glucose metabolism [49,50]. Acetate stimulates the growth of bacteria colonizing the mucus layer [51]. Mucin degradation products can also serve as nutritional resources for other bacteria [52]. *A. muciniphila* induces a specific T-cell response that increases the production of IgG1 antibodies in vivo [53]. However, there are significant individual differences in T-cell responses caused by *A. muciniphila.* Furthermore, microbial antigens from *A. muciniphila* induce the transformation of naive CD4^+^ CD44^-^Foxp3^-^T (Tn) cells into Treg lines [54]. *A. muciniphila* could also promote the biosynthesis of vitamin B12 [55].

**Fig. 1.**
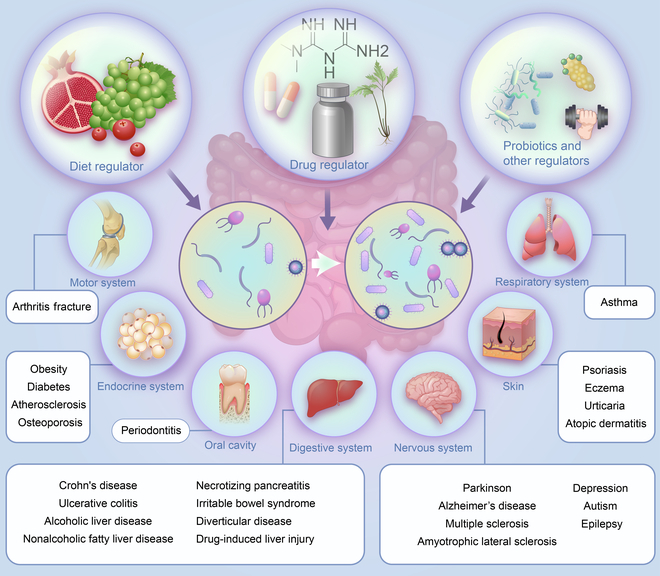
Changes in *A. muciniphila* abundance and regulators of *A. muciniphila*. Abnormal abundance of *A. muciniphila* can occur in the endocrine, nervous, digestive, respiratory, and motor systems. Under the influence of various factors, *A. muciniphila* abundance changes and affects the progression of these diseases.

*A. muciniphila* not only plays a role in maintaining normal physiological functions of the body but also is involved in the occurrence and development of endocrine system diseases, nervous system diseases, digestive system diseases, musculoskeletal system diseases, respiratory system diseases, and other diseases (Fig. 1) [56]. *A. muciniphila* has great potential to become one of the next generations of probiotics, to be applied to the diagnosis and treatment of human diseases, and to trigger a wave of intestinal microbial research.

## The Role of *A. muciniphila* in Disease

### Nonneoplastic disease

#### Endocrine system diseases

##### Obesity

In 2013, Everard et al. [57] found that the abundance of *A. muciniphila* decreases significantly in high-fat diet-induced and leptin-deficient obese mice. This study was the beginning of research on *A. muciniphila* and obesity. Increasing evidence has demonstrated an association between *A. muciniphila* and obesity. A study at Arizona State University found that healthy adolescent college freshmen with increased body mass index (BMI) and waist circumference (WC) have a lower abundance of *A. muciniphila* [58]*.* This phenomenon can also be observed in preschool children and pregnant women [59–61]. The abundance of *A. muciniphila* is correlated with the degree of obesity, history of bariatric surgery, and a variety of obesity markers [62–64]. In vivo results showed that the abundance of *A. muciniphila* is negatively correlated with body fat content, fat mass gain, and abdominal fat [65]. The *A. muciniphila* genome has a 3-gene operon in which tryptophanyl-tRNA synthase is related to waist-hip ratio and fat distribution [66,67]. Functionally, *A. muciniphila* inhibits the expression of PPARγ, C/EBPα, CD36, and other lipid marker genes and lipase genes in 3T3-L1 cells [68]. Moreover, pasteurized *A. muciniphila* reduces body weight, calorie intake, serum total cholesterol (TC) concentration, and blood glucose levels in high-fat diet-fed mice [68–71]. Pasteurized *A. muciniphila* increases energy expenditure and oxygen consumption in high-fat diet-fed mice. The fecal energy content of mice also increases significantly by the administration of pasteurized *A. muciniphila* [69]. In 2019, the results from a human trial confirmed the safety of *A. muciniphila* supplementation [72]. Oral supplementation with *A. muciniphila* significantly increased insulin sensitivity and reduced plasma TC levels, but the effect on obesity was not statistically significant.

**Fig. 2.**
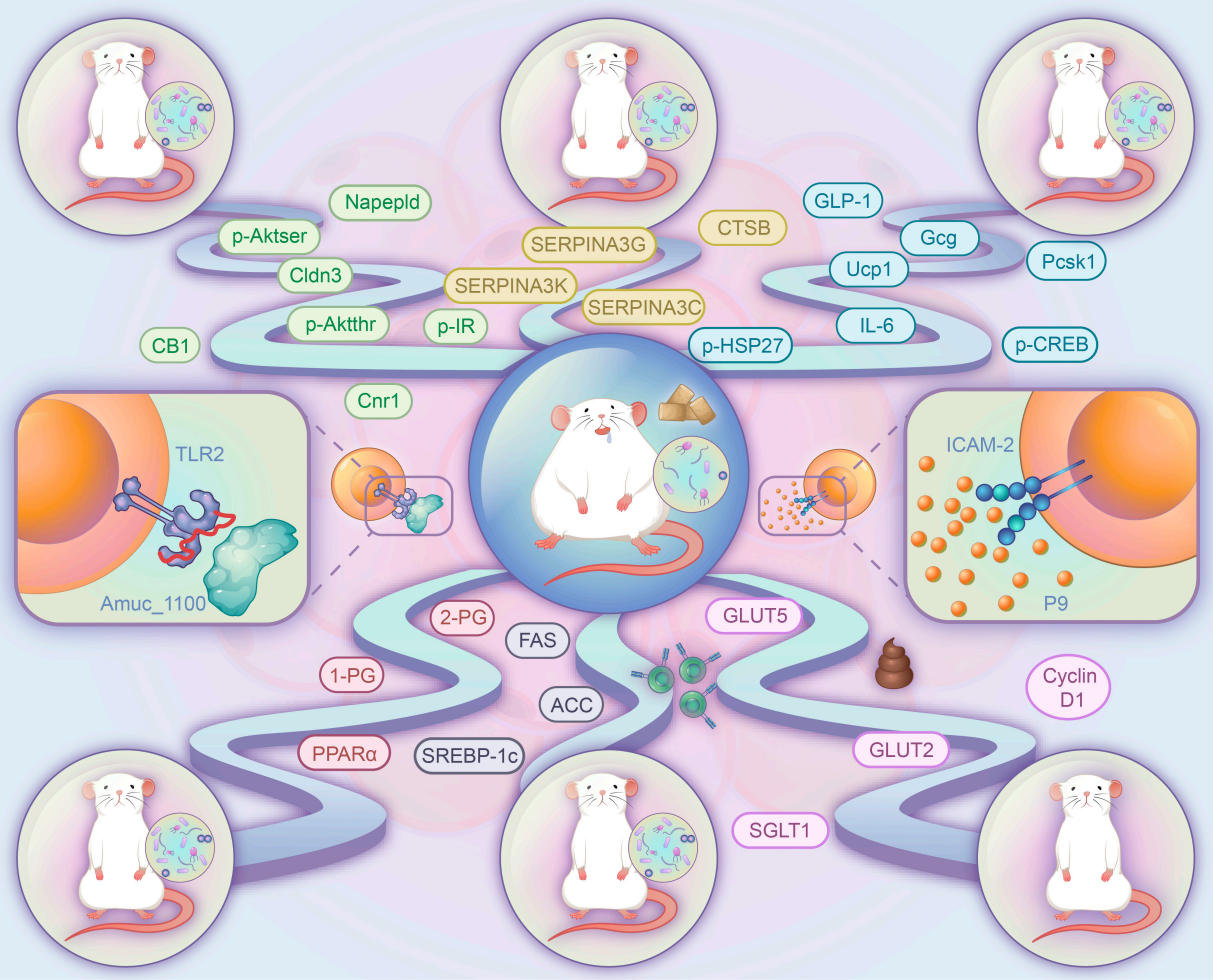
The regulatory mechanism of *A. muciniphila* in obesity. Amuc_1100 promotes p-Akt^thr^ and Akt^ser^ expression to enhance insulin sensitivity. Amuc_1100 improves intestinal permeability activation by regulating Cldn3 expression and the Cnr1/CB1 axis in vivo. Cldn3 expression regulated by Amuc_1100 also contributes to the improvement of intestinal permeability. P9 recognizes and binds to the ligand ICAM-2. P9 then induces GLP-1 secretion by promoting p-CREB and p-HSP27 expression levels. P9 promotes thermogenesis and improves obesity and glucose homeostasis in high-fat diet-fed mice by activating the GLP-1R signaling pathway and IL-6. *A. muciniphila* administration inhibits adipogenesis in adipocytes via the SERPINA3/CTSB axis. It also increases 2-pg and 1-pg levels in circulating blood and activates peroxisome proliferator-activated receptor alpha (PPARα) to regulate lipid metabolism. *A. muciniphila* reduces carbohydrate absorption by reducing GLUT2, GLUT5, and SGLT1 expression. *A. muciniphila* reduces the expression levels of the hepatic lipogenesis markers SREBP-1c, ACC, and FAS.

In 2016, researchers isolated the outer membrane protein (Amuc_1100) of *A. muciniphila*. The function of Amuc_1100 is not affected by pasteurization. Amuc_1100 specifically induces cellular immunity by activating TLR2 [73]. It also increases p-Akt^thr^ and p-Akt^ser^ levels to improve insulin sensitivity (Fig. 2). Additionally, Amuc_1100 activates Cnr1 and promotes CB_1_ expression to improve intestinal permeability in vivo. Cldn3 expression regulated by Amuc_1100 also contributes to the improvement in intestinal permeability [73]. Recently, scientists extracted an 84-kDa protein, named P9, from the secretions of *A. muciniphila*. P9 has a stronger effect than Amuc_1100 on improving obesity and glucose homeostasis [74]. The gastrointestinal hormone glucagon-like peptide 1 (GLP-1) can regulate energy balance and improve glucose homeostasis. The effect of P9 on GLP-1 secretion is 1,000 times stronger than that of Amuc_1100. After binding with its ligand intercellular adhesion molecule 2 (ICAM-2), P9 promotes thermogenesis and improves obesity and glucose homeostasis by activating the GLP-1R signaling pathway and interleukin-6 (IL-6). The mechanism associated with *A. muciniphila* has attracted the attention of many researchers investigating endocrine system diseases. The expression of hepatic lipogenesis markers (sterol regulatory element-binding protein 1c [SREBP-1c], acetyl CoA carboxylase [ACC], and fatty acid synthase [FAS]) is decreased after *A. muciniphila* administration [71]. *A. muciniphila* reduces carbohydrate absorption by decreasing the expression of glucose transporters such as glucose transporter 2 (GLUT2) and sodium-glucose linked transporter 1 (SGLT1) [69]. It also increases the levels of 2-PG and 1-PG and activates peroxisome proliferator-activated receptor alpha (PPARα) [75]. The cell lysate of *A. muciniphila* increases serine protease inhibitor A3G (SERPINA3G) expression and reduces lipogenesis in adipocytes [76]. Cathepsin B (CTSB) is a key molecule by which SERPINA3 inhibits adipogenesis. In addition, *A. muciniphila* improves the inflammatory response of brown adipose tissue, alleviates endotoxemia, and improves the barrier of the gut mucosa [70]. A higher abundance of *A. muciniphila* is associated with higher expression of sCD14 and IL-10 [77]. Both live *A. muciniphila* and pasteurized *A. muciniphila* significantly reduce the number of CD4+ Foxp3+ cells in vivo [71].

##### Diabetes

*A. muciniphila* abundance decreases in obese diabetic mice and obese patients with type 2 diabetes mellitus (T2DM) [57,66,78–83]. The abundance of *A. muciniphila* is negatively correlated with fasting blood glucose and glycosylated hemoglobin levels [84]. Oral administration of *A. muciniphila* decreases levels of liver glycogen and increases serum insulin levels and glucose tolerance [74,85]. However, increased *A. muciniphila* abundance after bariatric surgery is not associated with glucose homeostasis [62,64]. However, Amuc_1100 and P9 play important roles in the regulation of glucose homeostasis [73,74]. P9 improves GLP-1 secretion and glucose tolerance [74]. Interferon γ (IFNγ) could modulate the role of *A. muciniphila* in glucose tolerance [86]. Glucose tolerance is significantly improved in IFNγ-knockout mice. IFNγ decreases the abundance of *A. muciniphila* in the gut mucosa by promoting the expression of Irgm1 in vivo. Pasteurized *A. muciniphila* reduces carbohydrate absorption by downregulating GLUT2 and SGLT1 expression [69]. *A. muciniphila* also enhances FGF15/19 to stimulate glycogen synthesis by inhibiting βCDCA [83]. However, the findings by Qin et al. are contrary to all the above results regarding the variation of *A. muciniphila* abundance in T2DM. The results showed that increased *A. muciniphila* abundance is significantly upregulated in T2DM. The enrichment of *A. muciniphila* is related to mucin degradation, but the regulation of *A. muciniphila* on T2DM was not evaluated in the study [87].

The abundance of *A. muciniphila* in the gut is significantly reduced in patients with type 1 diabetes (T1D) [88]. *A. muciniphila* could decrease serum endotoxin levels and promote mucus secretion and the expression of Reg3γ in non-obese diabetic (NOD) mice [89,90]. *A. muciniphila* inhibits T1D development by decreasing TLR expression, reducing monocyte infiltration of islet cells, and increasing the number of regulatory Foxp3^+^ Treg cells.

###### Atherosclerosis

In cases of obesity or diabetes, lipid metabolism is usually abnormal. Li et al. [91] found that *A. muciniphila* abundance is negatively correlated with the severity of atherosclerosis. A higher abundance of *A. muciniphila* is associated with lower levels of soluble tumor necrosis factor receptor II (sTNFR II). Camp-responsive element-binding protein H (CREBH) is a lipid regulatory transcription factor that binds to the endoplasmic reticulum. Increased intestinal *A. muciniphila* abundance reduces the production of chylomicron and very-low-density lipoprotein in CREBH-null mice [92]. *A. muciniphila* significantly reduces apoB100 and plasma triglyceride (TG) levels in CREBH-null mice but does not affect wild-type mice. *A. muciniphila* also promotes the clearance of plasma TGs and chylomicrons by increasing the levels of LDL receptors in CREBH-null mice. Apoe^−/−^ mice and Apoe*3Leiden mice. CETP mice are commonly selected models for studying lipid metabolism and atherosclerosis. Fasting plasma TC and TG levels decrease in hyperlipidemic E3L CETP mice after oral administration of *A. muciniphila* for 4 weeks [93]. However, oral administration of *A. muciniphila* has no significant effect on serum TC, total TG, low-density lipoprotein, or high-density lipoprotein levels in Apoe^−/−^ mice [91]. Whether *A. muciniphila* regulates the progression of atherosclerosis by regulating lipid metabolism remains to be further studied. Additionally, *A. muciniphila* regulates local and systemic immune responses. *A. muciniphila* affects the composition of immune cells in mesenteric lymph nodes and promotes the expression of CD86 on dendritic MHCII and B cells. The levels of macrophages, macrophage markers (F4/80), and inflammatory factors (IL-1β, tumor necrosis factor α [TNF-α], and monocyte chemoattractant protein 1 [MCP-1]) in the aorta and plasma of Apoe^−/−^ mice decreased after oral administration of *A. muciniphila*. *A. muciniphila* protects against atherosclerosis by regulating systemic and vascular inflammation [91,93].

##### Osteoporosis

Most studies suggest that *A. muciniphila* is beneficial, but some have found that *A. muciniphila* promotes osteoporosis [94–96]. *A. muciniphila* is positively correlated with bone formation markers, 25-OH-D, and bone resorption markers [94]. *A. muciniphila* promotes bone resorption by increasing serum parathyroid hormone levels and serum amyloid A3 levels. Treg cells have anti-inflammatory effects that reduce bone loss. The number of Treg cells in the bone marrow and mesenteric lymph nodes decreased after pasteurized *A. muciniphila* administration.

#### Nervous system diseases

##### Neurodegenerative diseases

Neurodegenerative diseases such as Parkinson’s disease (PD), Alzheimer’s disease (AD), amyotrophic lateral sclerosis (ALS), and multiple sclerosis (MS) have become major public health problems, particularly for elderly individuals. Changes in *A. muciniphila* abundance can contribute to the occurrence and development of a variety of neurodegenerative diseases. Unlike the concordant role of *A. muciniphila* in most diseases, the role of *A. muciniphila* is changing in neurodegenerative diseases. *A. muciniphila* abundance is significantly downregulated in AD and ALS, while the abundance of *A. muciniphila* is upregulated in MS and PD.

Studies linking gut microbes to AD date back to 2016. Changes in the gut flora promote the progression of brain inflammation and amyloid-β (Aβ) protein deposition. *A. muciniphila* abundance is obviously downregulated in AD [97]. AD mice and APP/PS1 mice are common mouse models in AD research. *A. muciniphila* administration improves spatial learning and memory by reducing Aβ plaque deposition and Aβ40–42 levels in APP/PS1 mice [98]. *A. muciniphila* administration also promotes hippocampal glial cell proliferation and the expression of proinflammatory cytokines to improve neural development and synaptic plasticity. Early feeding of a high-fat diet impairs the learning and memory ability of AD mice and reduces the abundance of *A. muciniphila*. *A. muciniphila* administration improves glucose metabolism, the intestinal barrier, and lipid metabolism in mouse models of AD [98]. *A. muciniphila* also promotes the expression of UCP1 and increases thermogenesis in brown adipose tissue of AD mice. Although most studies suggest that *A. muciniphila* is a protective factor against AD, Khedr et al. observed the upregulation of *A. muciniphila* in AD patients. Further research is needed to explore the reasons for this refutation [99].

The development of ALS is also regulated by the gut microbiome. In vivo experiments showed that motor function was significantly impaired by treatment with antibiotics. The decreased *A. muciniphila* abundance is found in ALS. *A. muciniphila* administration can significantly improve the symptoms of ALS. Nicotinamide, a metabolite of *A. muciniphila*, plays a key role in the treatment of ALS. Nicotinamide supplementation improves motor function in vivo. ALS patients have lower levels of nicotinamide in the blood and cerebrospinal fluid than control individuals [100].

MS is an autoimmune-related neurodegenerative disease. Gut microbes regulate immune responses to cause neurodegenerative changes and the development of MS [101]. Experimental autoimmune encephalomyelitis is exacerbated in mice after stool transplantation from MS patients [102]. Increased *A. muciniphila* abundance was also found in MS patients compared with paired household healthy controls [103]. 16S rRNA sequencing analysis showed that *A. muciniphila* abundance is significantly higher in twins with MS than in healthy twins [101,102]. *A. muciniphila* stimulates peripheral blood mononuclear cells to differentiate into TH1 cells, promoting an inflammatory T-lymphocyte response [102].

**Fig. 3.**
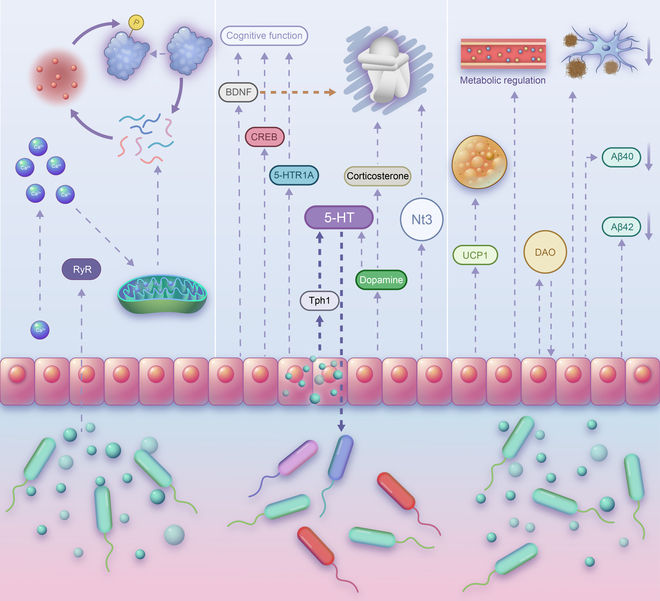


The aggregation of α-synuclein is a key process in the pathogenesis of PD. Although this toxic aggregation occurs almost throughout the central system, increasing evidence showed that α-synuclein aggregation is found in enteric nerves before intracerebral aggregation of α-synuclein. These findings suggest that PD may originate within the gut. Some studies suggest that *A. muciniphila* abundance is significantly increased in PD, which contrasts with previous indications that *A. muciniphila* has beneficial effects in most diseases [104,105]. *A. muciniphila*-conditioned medium (CM) leads to ROS production and α-synuclein aggregation in enteroendocrine cells by increasing intracellular Ca^+^ levels (Fig. 3) [105]. However, the methods of these studies are limited. More complex models should be adopted in the study of *A. muciniphila*’s role in PD.

The regulatory mechanism of *A. muciniphila* in neurodegenerative diseases. Activation of RyR by *A. muciniphila*-conditioned medium increases the intracellular Ca^+^ concentration and promotes the misfolding of α-synuclein via ROS in Parkinson’s disease. In Alzheimer’s disease, *A. muciniphila* inhibits Aβ plaque deposition and reduces the expression levels of Aβ40 and Aβ42 to improve learning and memory in APP/PS1 mice. *A. muciniphila* also activates UCP1 to promote brown adipose tissue thermogenesis in AD mice. Glucose metabolism and the gut barrier in APP/PS1 mice are also regulated by *A. muciniphila*. *A. muciniphila* exerts an antidepressant effect by regulating serum corticosterone, dopamine, BDNF, CREB, NT3, and other depression-related molecules. *A. muciniphila* increases intestinal 5-HT levels in CUMS mice by activating the rate-limiting enzyme TPH1 for 5-HT synthesis.

##### Depression

The abundance of *A. muciniphila* decreases in depressed mice and Wistar-Kyoto (WKY) rats [106–108]. *A. muciniphila* abundance is positively correlated with the expression of MUC2 [106]. Oral administration of *A. muciniphila* significantly improves depression-like behavior in vivo [108,109]. *A. muciniphila* promotes the repair of the gut barrier and reduces depression-related colitis damage [106]. *A. muciniphila* decreases serum concentrations of corticosterone and increases dopamine and brain-derived neurotrophic factor (BDNF) levels in the hippocampus [109,110]. Amuc_1100 exerts antidepressant effects by regulating the gut flora, serum metabolites, and BDNF (Fig. 3). Extracellular vesicles (EVs) from *A. muciniphila* promote neurotrophic factor expression [108].

##### Autism

Wang et al. [111] found that the abundance of *Bifidobacterium* and *A. muciniphila* in autistic children is significantly reduced. In children with autism, the intestinal mucus layer becomes thinner. Reduced *A. muciniphila* abundance may contribute to the development of autism by reducing mucus barrier function and increasing the permeability of the gut mucosa. Fecal bacteria transplantation increases *A. muciniphila* abundance in FMR1-knockout mice with autism and improves cognitive dysfunction and social novelty preference [112]. However, fecal bacteria transplantation entails transplantation of the whole microbiome, and the role of *A. muciniphila* in the treatment of autism still needs further study.

##### Epilepsy

Ketogenic diets (KDs) mediated by gut microbes can be used to treat refractory epilepsy. *A. muciniphila* abundance in the gut of mice with 6-Hz-induced seizures increases significantly due to KDs [113,114]. The antiepileptic effect of KDs disappears after either aseptic feeding or antibiotic treatment. Simultaneous supplementation with *A. muciniphila* and Parabacteroides increases the threshold of epileptic seizures and rescues the antiepileptic effect of KDs in vivo. *A. muciniphila* and Parabacteroides increase GABA levels and the GABA/glutamate ratio in the hippocampus. *A. muciniphila* also decreases the activity of γ-glutamyl transpeptidase and inhibits γ-glutamylation. The combination of *Bifidobacterium* and *A. muciniphila* administration reduces the cytotoxicity induced by carbamazepine and lamotrigine in vitro [115].

#### Digestive system diseases

##### Inflammatory bowel disease (IBD)

*A. muciniphila* abundance is decreased in patients with Crohn’s disease or ulcerative colitis and multiple animal models of colitis [45,116–121]. *A. muciniphila* abundance is negatively correlated with colitis activity and inflammation score but positively correlated with the percentage of sulfated colonic mucins in the mucus gel layer [122]. Pasteurized *A. muciniphila*, *A. muciniphila*-derived EVs, Amuc_1100, and Amuc_2109 can significantly reduce colitis activity; alleviate colonic tissue damage and splenomegaly; and alleviate colonic infiltration by macrophages, CD8^+^ cytotoxic T lymphocytes (CTLs), inflammatory factors, TNF-α, IFNγ, and other infiltrating factors [22,106,123,124]. However, the components of EVs derived from *A. muciniphila* are complex, and future research should confirm the role of EVs in the pathogenesis of colitis. Amuc_2109 significantly decreases malondialdehyde (MDA) levels induced by dextran sulfate sodium (DSS) and increases glutathione (GSH) and superoxide dismutase (SOD) activities [124]. In the ipEC-J2 cell inflammatory model, *A. muciniphila* administration can also alleviate the inflammatory response of intestinal epithelial cells by regulating the expression of the phosphatidylinositol 3-kinase (PI3K) upstream receptor genes [125]. *A. muciniphila* inhibits apoptosis by inhibiting the expression of key genes in calcium signaling pathways and cell cycle signaling pathways [125]. NOD-like receptor family pyrin domain-containing 3 (NLRP3) is a relatively well-studied member of the NLR family and is a key molecule in pathogen recognition and cellular immunomodulation. In vitro and in vivo studies showed that NLRP3 mRNA and protein expression increases with increasing *A. muciniphila* abundance [45]. The anticolitis effect of *A. muciniphila* weakens after NLRP3 knockout. *A. muciniphila* alleviates DSS-induced colitis by activating NLRP3.

Tryptophan and its metabolites (5-hydroxytryptamine [5-HT], kynurenine, and indole derivatives) also play an important role in the development of colitis. *A. muciniphila* is significantly correlated with tryptophan metabolites. Pasteurized *A. muciniphila* promotes the expression of IA and IAA to activate AhR signaling and reduce colon inflammation [126]. *A. muciniphila* can increase serotonin transporter expression and increase the bioavailability of 5-HT in the gut [127]. The inhibitory effect of *A. muciniphila* on colitis is strain specific. *A. muciniphila* FSDLZ36M5-treated mice are significantly smaller in body weight and have a shorter colon length, while the levels of proinflammatory cytokines (TNF-α, IL-1β, and IL-6) increase and the levels of anti-inflammatory cytokines (IL-10) decrease [128]. However, *A. muciniphila* FSDLZ39M14, ATCC BAA-835, and FSDLZ20M4 did not significantly alleviate colitis in mice, and *A. muciniphila* FSDLZ20M4 even aggravated gut injury in mice. Both strain ATCC and strain 139 improve chronic colitis in mice, and strain ATCC has a stronger effect [129].

##### Nonalcoholic fatty liver disease (NAFLD) and alcoholic liver disease (ALD)

*A. muciniphila* affects liver fat metabolism by regulating the expression of genes related to fat synthesis and different inflammatory factors, thereby preventing nonalcoholic fatty liver disease (NAFLD). *A. muciniphila* also mediates the effect of a variety of drugs used for the treatment of NAFLD, such as Si Miao Formula [130], MDG [131], and Bofutsushosan [132]. MDG alleviates obesity-induced liver damage and steatosis by increasing *A. muciniphila* abundance [131]. *A. muciniphila* abundance decreases in both mice and patients with NAFLD [132,133]. Higher *A. muciniphila* abundance is associated with lower concentrations of aspartate aminotransferase (AST), alanine aminotransferase (ALT), TC, and TG in serum [131]. The combination of quercetin and *A. muciniphila* can improve NAFLD [134]. *A. muciniphila* alleviates hepatic steatosis by regulating lipid oxidation and bile acid metabolism [135]. L-aspartic acid, a key component of the activator of the liver kinase B1 (LKB1)–AMP-activated protein kinase (AMPK) axis, promotes the expression of energy metabolism regulators and mitochondrial complexes in the liver [136]. *A. muciniphila* activates the LKB1–AMPK axis by increasing liver L-aspartic acid levels. Morrison et al. [135], however, found that although pasteurized *A. muciniphila* can improve intestinal permeability and mesenteric adipose tissue expansion, it has no significant effect on the development of NAFLD.

The abundance of *A. muciniphila* in ASH patients is significantly lower than that in nonobese healthy individuals [137]. Lower *A. muciniphila* abundance is associated with more serious liver lesions. Oral administration of *A. muciniphila* can alleviate alcoholic liver disease (ALD) [137]. Wild-type mice were fed the Lieber–DeCarli diet for 15 days, and then *A. muciniphila* was administered on day 10. *A. muciniphila* administration led to decreases in ALT, IL-1β, and TNF-α levels and MPO^+^ neutrophil infiltration. KRQKYD enhances the gut barrier and inhibits the inflammatory response mediated by *A. muciniphila*, thereby reducing susceptibility to ALD [137–139]. SREBP1c is a key component of liver adipogenesis. KRQKYD reduces SREBP1c expression by activating the NRF2/HO-1 antioxidant defense system and thereby inhibits lipid synthesis in the liver [140].

##### Other digestive system diseases

*A. muciniphila* abundance is also associated with necrotizing pancreatitis, irritable bowel syndrome (IBS), diverticular disease, and drug-induced liver injury. The abundance of *A. muciniphila* is significantly higher in patients with necrotizing pancreatitis than in healthy controls [141]. *A. muciniphila* abundance in symptomatic uncomplicated diverticular disease (SUDD) and asymptomatic diverticulosis is significantly higher than that in healthy controls [142]. Increased abundance of *A. muciniphila* can significantly improve the pain intensity of IBS [143]. *A. muciniphila* can significantly improve APAP-induced liver injury and reduce serum ALT and AST levels [144]. Mechanistically, *A. muciniphila* can significantly reduce GSH and SOD levels; inhibit liver infiltration by macrophages and neutrophils; and contribute to the release of IL-1β, IL-2, IL-6, and TNF-α. *A. muciniphila* regulates Bcl-2 and Bax expression to reduce apoptosis by activating the PI3K/Akt signaling pathway [144]. *A. muciniphila* also has protective effects on liver injury induced by HFD/CCl4 and concanavalin A, but these studies focused only on mice [145–147]. Owing to differences in the immune system and microbial composition between mice and humans, further studies are needed.

#### Diseases of the musculoskeletal system

##### Arthritis

*A. muciniphila* abundance is significantly lower in arthritis patients and arthritis animal models than in healthy controls [148–151]. In HLA-B27/β2 m+ rats, higher *A. muciniphila* abundance is associated with higher expression levels of inflammatory cytokines (IFNγ, IL-17A, and IL-23) and a higher incidence of spinal arthritis [148]. Ankle arthritis is aggravated when fecal microbes from spinal arthritis are transplanted into germ-free KRN/B6xNOD mice. *A. muciniphila* abundance is also negatively correlated with ankle swelling [150]. These studies have some limitations, such as inaccurate selection of the control group, and their results need further experimental verification. The role of chondroitin sulfate (CS) in the treatment of arthritis is controversial. Wang et al. [151] found that changes in gut flora play a decisive role in the treatment of osteoarthritis by CS. If the abundance of sulfatase-secreting bacteria (SSB) and sulfate-reducing bacteria (SRB) decreases while the abundance of *A. muciniphila* increases, then CS can improve OA. If *A. muciniphila* has a disadvantage in competition with SSB and SRB, CS can aggravate OA. This finding has stimulated the development of CS-related cocktails. The combination of CS with probiotics might inhibit the progression of arthritis.

##### Fracture

*A. muciniphila* can also promote revascularization and thus contribute to fracture healing. Administration of *A. muciniphila* reduces the amount of mineralized bone volume and total callus volume in a fractured segment [152]. Although *A. muciniphila* administration also changes the gut microbial community, such as reducing the abundance of γ-proteobacteria, the above effects of *A. muciniphila* still exist after pretreatment with antibiotics. *A. muciniphila* can improve intestinal barrier function in mice, inhibit the systemic inflammatory response, and promote the formation of type H vessels to promote fracture healing [152].

#### Respiratory system diseases

##### Asthma

*A. muciniphila* and *Faecalibacterium prausnitzii* abundances decrease significantly in asthmatic patients [153]. Lower abundances of *A. muciniphila* and *F. prausnitzii* are associated with greater severity of asthma [154]. In vivo studies show that *A. muciniphila* significantly reduces airway hyperresponsiveness and airway inflammation. *A. muciniphila* administration significantly decreased the number of BAL eosinophils and IFNγ^+^ CD4 T cells, while the number of IL-10^+^Foxp3^+^ lymphocytes was increased by *A. muciniphila* administration. After oral administration of *A. muciniphila*, airway hyperreactivity to acetylcholine is significantly reduced in vivo [153]. Resveratrol (RES) attenuates allergic asthma and increases pulmonary *A. muciniphila* abundance in ovalbumin-exposed mice [155]. However, whether RES plays a role in lung *A. muciniphila* mediation needs to be confirmed.

#### Other diseases

##### Cutaneous disorders

Recent studies on the skin microbiome show that skin-related diseases, such as psoriasis, eczema, urticaria, and atopic dermatitis in children, are associated with changes in *A. muciniphila* abundance. *A. muciniphila* abundance decreases in chronic urticaria but increases in infantile eczema [156–159]. The changes in *A. muciniphila* abundance in psoriasis are controversial [156,160]. A significant increase in *A. muciniphila* abundance in patients with psoriasis is contrary to the findings of some research teams [156,158,160]. However, *A. muciniphila* abundance is also a marker of psoriasis biotherapy. Changes in *A. muciniphila* abundance are most significant in patients who receive systemic biotherapy compared to controls [161]. However, this study did not sample individual patients at different time points, and the experimental results are susceptible to many potentially confounding factors, such as the effects of age, sex, and severity of disease. The combination of *Bifidobacterium breve*, *Bifidobacterium pseudocatenulatum*, *Bifidobacterium adolescentis*, *Escherichia coli*, *F. prausnitzii*, and *A. muciniphila* can be used to identify whether pediatric atopic dermatitis is related to food allergy [162].

##### Oral diseases

*A. muciniphila* abundance decreases in patients with periodontitis [163]. *A. muciniphila* is also present in the oral cavity of healthy individuals and disappears completely in the progression of periodontitis [164]. *A. muciniphila* is effective in treating periodontitis caused by *Porphyromonas gingivalis*. In the experimental periodontitis model, *A. muciniphila* or Amuc_1100 reduces the inflammatory response and osteoclast activity in the interdental spaces, thereby reducing alveolar bone loss [165,166]. *A. muciniphila* antagonizes the elevation of IL-12 levels and the decrease in IL-10 expression induced by *P. gingivalis* in bone marrow-derived macrophages. *A. muciniphila* or Amuc_1100 increases the ratio of M2 macrophages/M1 macrophages during *P. gingivalis* infection and recruits Th1 cells into gingival tissue by upregulating CXCL10 [167]. *A. muciniphila* administration promotes the expression of intercellular adhesion molecule 1 (ICAM-1) and tight binding molecules (ZO-1) in gingival epithelial cells (TIGK). However, only oral administration, not gastric gavage, prevents periodontitis caused by *P. gingivalis* [166]*.* Pasteurized *A. muciniphila* is a very promising drug for the treatment of periodontitis.

##### Aging

*A. muciniphila* administration prolongs the lifespan and reduces the incidence of age-related complications in vivo [168,169]. After *A. muciniphila* supplementation, the expression of Reg3g and Tff3 in the ileum of Ercc1-/Δ7 mice increases, which promotes barrier repair of the mucosal layer and wound healing [170]. Studies on senescence-related metabolites show that *A. muciniphila* can increase polyamine, 2-hydroxybutyrate, SCFA, and bile acid levels in the gut [169].

### Neoplastic disease

**Fig. 4.**
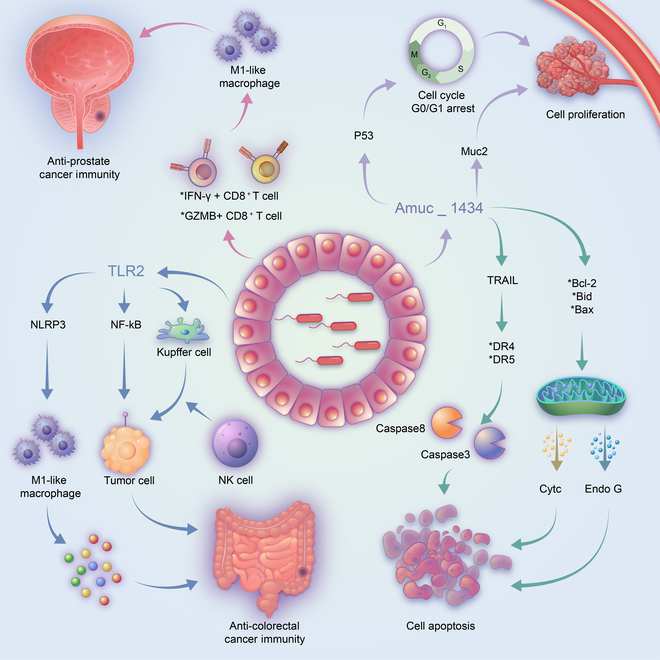
The regulatory role of *A. muciniphila* in neoplastic disease. Amuc_1434 induces G0/G1 cell cycle arrest by promoting p53 expression in colorectal cancer. Amuc_1434 inhibits the proliferation of LS174T cells through regulation of Muc2. Amuc_1434 activates death receptors and mitochondrial apoptosis pathways to promote apoptosis in LS174T cells. In colorectal cancer, *A. muciniphila* induces M1-like macrophage polarization by regulating the TLR2/NLRP3 and NF-κB pathways; activated NLRP3 in Kupffer cells promotes the recruitment of NK cells to kill tumor cells. *A. muciniphila* extracellular vesicles enhance the anti-prostate cancer immune response of cytotoxic T lymphocytes by increasing the proportion of GZMB^+^CD8^+^ T cells and IFNγ^+^CD8^+^ T cells.

Differences in the fecal microbiota exist in many cancers and are risk factors for cancer development [171]. *A. muciniphila* can inhibit the progression of lung cancer and prostate cancer [172,173]. Alterations in *A. muciniphila* abundance are controversial in patients with colorectal cancer (CRC) [174–176]. Functionally, *A. muciniphila* administration can inhibit cell proliferation in CRC [22,174,177]. The inhibitory effect of Amuc_1434, a protein derived from *A. muciniphila*, on CRC cell proliferation and the cell cycle is enhanced with increasing Amuc_1434 concentration [177]. Amuc_1434 induces G0/G1 arrest by promoting p53 expression (Fig. 4). Muc2 is a key molecule by which Amuc_1434* inhibits proliferation. Amuc_1434 activates the death receptor and mitochondrial apoptotic pathways to promote apoptosis by regulating caspase 3, caspase 8, and the tumor necrosis factor-related apoptosis-inducing ligand (TRAIL). Further in vivo experiments confirmed the inhibitory effect of Amuc_1434 on CRC. The immune system is the medium of the *A. muciniphila*–tumor interaction. *A. muciniphila* promotes the activation of M1-like macrophages and the expression of related cytokines (IL-23, TNF-α, and IL-27) [174]. The NLRP3 pathway is a key pathway for the polarization of macrophages. *A. muciniphila* abundance is negatively correlated with NLRP3 and TLR2 expression. *A. muciniphila* promotes the polarization of M1-like macrophages and inhibits the progression of CRC by regulating the TLR2/NLRP3 and NF-κB pathways. Activated NLRP3 promotes the recruitment of NK cells. CTLs are the main cellular mediators of anti-CRC immunity. *A. muciniphila* and Amuc_1100 recruit CTLs and increase the percentage of CTLs in the mesenteric lymph nodes and colon by promoting the secretion of chemokines [22]. However, the interaction between macrophages and CTLs in the progression of colon cancer remains to be studied. Similarly, in vitro and in vivo experiments show that *A. muciniphila* EVs increase the number of M1 macrophages in prostate cancer tissues. CM from *A. muciniphila*-EV-treated macrophages inhibits PCa cell proliferation and invasion [173]. *A. muciniphila* EVs induce a CTL anti-prostate cancer immune response by increasing the proportion of GZMB^+^ and IFNγ^+^CD8^+^ T cells [173] (Fig. 4). EVs carry several immunogens that may affect human physiological functions. Researchers further explored the safety of *A. muciniphila* EVs. *A. muciniphila* EVs have no obvious toxicity to vital organs, such as the kidney, liver, and normal prostate cell lines. *A. muciniphila* EVs are well tolerated in vitro and in vivo. *A. muciniphila* EVs are a promising treatment for prostate cancer.

*A. muciniphila* is a key regulator of many anticancer drugs and affects the efficacy of antitumor therapy. Oxaliplatin, fluorouracil, and FOLFOX (oxaliplatin, fluorouracil, and calcium folinate) are commonly used as first-line chemotherapies for colon cancer. Hou et al. [178] found a significant increase in *A. muciniphila* abundance in colon cancer patients treated with FOLFOX. Increased abundance of *A. muciniphila* is associated with better prognosis in colon cancer patients treated with FOLFOX. In lung cancer, *A. muciniphila* administration combined with cisplatin (CDDP) significantly inhibits tumor growth and improves lung cancer tissue morphology at the pathological level in Lewis lung cancer mice [179]. Mechanistically, *A. muciniphila* administration combined with CDDP downregulates the expression of Ki-67, p53, and FasL. Immune checkpoint inhibitors (ICIs) that target the PD-1/PD-L1 immune checkpoint significantly improve patient outcomes. However, ICI drug resistance is a major problem in cancer diagnosis and treatment. It is very important to explore the mechanism of ICI resistance and to identify biomarkers for predicting therapeutic effects. Tumor patients taking antibiotics are resistant to PD-1 inhibitors and have poor prognoses [180]. *A. muciniphila* abundance increases significantly in patients with epithelial tumors who have a good response to ICI treatment. *A. muciniphila* administration improves the therapeutic response to PD-1 inhibitors in vivo [180]. *A. muciniphila* abundance is positively associated with the objective response rate and overall survival rate in patients with advanced non-small cell lung cancer (NSCLC) who receive ICI treatment [181,182]. *A. muciniphila* abundance can be used as a marker to predict the response to ICI treatment in patients with NSCLC. HYR-2 may play an anti-lung cancer role by inhibiting the protein expression of PD-L1 and increasing *A. muciniphila* abundance [183]. Abiraterone acetate (AA) is an androgen biosynthesis inhibitor targeting CYP17, and it also has therapeutic effects on androgen-independent prostate cancer. The mechanism of this phenomenon was explored by Daisley et al. [184] They found a significant increase in *A. muciniphila* abundance in the gut flora of prostate cancer patients treated with ADT^+^ (androgen deprivation therapy) AA. Regulating the intestinal ecosystem to improve cancer treatment is a promising research direction.

## Regulators of *A. muciniphila* Abundance

*A. muciniphila* is involved in the occurrence and development of a variety of human diseases, even cancer. It is a shining star among gut bacteria. Increasing *A. muciniphila* abundance may inhibit or even reverse disease progression. Though *A. muciniphila* uses mucin secreted by the intestinal mucosa as its sole source of energy, Dietary, drug-derived compounds and some gut microbes provide a microenvironment for *A. muciniphila* growth and could regulate *A. muciniphila* abundance. To obtain a more detailed and comprehensive understanding of *A. muciniphila* regulators and promote the clinical application of *A. muciniphila*, we summarize the relevant literature on *A. muciniphila* regulators (Fig. 1).

### Dietary regulators

Dietary intervention is the main way to regulate the abundance of gut microbes. Numerous studies have demonstrated that dietary regulators play an important role in the regulation of *A. muciniphila* abundance. Polyphenols include flavonoids, phenolic acids, stilbenes, and lignans. Dietary polyphenols, such as grape polyphenols [185], tea polyphenols [186,187], cranberry extract [188], pomegranate ellagic tannins [189], and black raspberry [190], are the most commonly studied substances regulating *A. muciniphila* abundance. Pomegranate extract, grape procyanidins, cranberry extract, and green tea polyphenols all significantly increase *A. muciniphila* abundance, thereby improving metabolism and intestinal inflammation. Grape polyphenols can reduce obesity, glucose intolerance, and oxidative stress caused by increased *A. muciniphila* abundance [191–193]*.* Cranberry extract promotes liver lipid metabolism and reverses insulin resistance by upregulating *A. muciniphila* abundance [194]. Mechanically, cranberry extract improves the gut barrier by enhancing mucin secretion and promotes *A. muciniphila* growth [195]. The mechanism of other polyphenols in upregulating *A. muciniphila* abundance remains unclear. Further experiments are needed to investigate this. The cometabolism of epigallocatechin-3-gallate, mucin, and glucose effectively promotes the growth of *A. muciniphila* in vitro [187]. These studies suggest that the gut–liver axis is the main target of dietary polyphenols. The antimicrobial activity of polyphenols and *A. muciniphila*’s tolerance to polyphenols may give *A. muciniphila* a competitive advantage in the gut. However, not all polyphenols are effective or equally effective in humans and animals. Oligosaccharides are also important regulators of *A. muciniphila*. Stachyose is used by probiotics in the gut. Both low-dose and high-dose stachyose can improve the abundance of *A. muciniphila* in the gut. High-dose stachyose can also promote the proliferation of bifidobacteria [196]*.* By regulating the gut microbiota, stachyose increases goblet cell number and tight junction protein expression. KDs increase the threshold of epileptic seizures by increasing *A. muciniphila* abundance in the gut of mice [114]. Fermentable oligosaccharides, disaccharides, monosaccharides, and polyols (FODMAP) diets also affect *A. muciniphila* abundance [197]. *A. muciniphila* abundance in people who ate a high-FODMAP diet was found to be significantly higher than that in a low-FODMAP diet group. *A. muciniphila* and *Lactobacillus* abundance were higher in the cecal contents of mice fed fish oil than in those fed lard [198,199]. Capsaicin (CAP) is the main pungent ingredient in chili peppers. Shen et al. [200] found an association between CAP concentration and *A. muciniphila* abundance. High concentrations of CAP (20 or 200 μg/ml) inhibited the growth of *A. muciniphila*, but low concentrations did not.

### Drug regulators

Metformin also plays an important role in the regulation of gut microbiota. Metformin can increase *A. muciniphila* abundance and decrease the abundance of Clostridiaceae 02d06 and Firmicutes [41,89,201]. The enhancement of gut barrier mediated by *A. muciniphila* may further promote the antidiabetic effect of metformin. Depletion of gut microbes using a combination of antibiotics (carbenicillin, metronidazole, neomycin, and vancomycin) eliminates metformin’s regulatory effects on glucose homeostasis [202]. The combination of metformin and mannan (MOS) improves the antidiabetic effect of metformin [203]. Metformin enhances the mucus barrier and anti-inflammatory effects by regulating the gut microbiota and increasing *A. muciniphila* abundance, thereby alleviating ulcerative colitis [204]. Early application of vancomycin can increase the abundance of *A. muciniphila* and Proteobacteria and reduce the incidence of diabetes [205]. OPS-2071 is a new quinolone antibacterial agent. The broad-spectrum antibacterial activity of OPS-2071 and its low bacteriostatic effect on *A. muciniphila* give *A. muciniphila* an advantage in gut colonization [206].

Long-term use of atorvastatin leads to gut microbiome disorders and a decrease in *A. muciniphila* abundance, thereby promoting weight gain and glucose tolerance [207]. Inulin and butyrate supplementation significantly increased *A. muciniphila* abundance and the expression levels of TNF-α, Hs-CRP, and MDA [208]. Hyaluronic acid–bilirubin nanomedicine (HABN) is a nanodrug targeting colitis via the HA–CD44 interaction. HABN regulates the distribution of gut flora and significantly increases the abundance of *A. muciniphila* [209]. A variety of herbs can also regulate *A. muciniphila* and inhibit disease progression. Berberine (BBR) is an extract from *Coptis chinensis* and *Phellodendri chinensis* that is used to treat various cardiovascular and metabolic diseases [210]. By stimulating the secretion of intestinal mucus, BBR indirectly changes the gut flora of mice in a dose-dependent and time-dependent manner and increases the abundance of *A. muciniphila* [211]*.* Supplementation with rhubarb extract can change the relative abundance of *A. muciniphila*, Parabacteroides, and Erysipelatoclostridium, among which *A. muciniphila* is the most affected [212,213]. Rhubarb extract maintains the gut barrier by promoting the interaction between *A. muciniphila* and Reg3y and alleviates alcohol-induced liver injury. Puerarin improves weight gain and glucose homeostasis by regulating the gut microbiota, particularly *A. muciniphila* [214]. In addition, other herbs, such as Panax notoginseng saponins, Bofutsushosan, the total flavone of Abelmoschus manihot, and Huoxue Yiqi Recipe-2 (HyR-2), modulate *A. muciniphila* abundance. They improve metabolism and even anticancer activity by affecting *A. muciniphila* [183,215–217].

### Probiotic regulators

The gut microbes interact with each other to influence the distribution and composition of the community. *Lactobacillus acidophilus* LA5 increases *A. muciniphila* abundance in the colon of mice [218]. The regulatory effect of *L. acidophilus* LA5 on *A. muciniphila* has not been confirmed in vitro. The acidifying property of *L. acidophilus* LA5 may be one reason that it promotes *A. muciniphila* growth. Alard et al. [219] showed that the *B. animalis* subsp. *lactis* strain can increase *A. muciniphila* abundance, while *L. rhamnosus* has the opposite effect. The effect of *B. animalis* subsp. *lactis* on *A. muciniphila* may be related to the increase in SCFA levels [219]. Exogenous *A. muciniphila* supplementation is the most direct way to regulate *A. muciniphila*. Kriebs [220] demonstrated the safety and efficacy of injecting *A. muciniphila* into humans in the treatment of metabolic syndrome.

*A. muciniphila* is difficult to culture and preserve. It is necessary to study suitable and convenient storage methods for *A. muciniphila*. EGCG-modified sodium alginate microcapsules can significantly improve the survival rate of *A. muciniphila* [221]. Dark chocolate, as a microencapsulated *A. muciniphila* carrier, can also maintain the survival of *A. muciniphila* for a long time [222]. This protective effect persists even in an in vitro astrointestinal model. Dark chocolate-containing microcapsules are expected to be an alternative carrier for *A. muciniphila* administration. However, the effect of chocolate on *A. muciniphila* and the appropriate concentration of *A. muciniphila* still need to be further explored.

### Other regulators

Secondary bile acids are produced by the biotransformation of gut microbes and are closely related to the abundance of *A. muciniphila* [223]. Most bile acids, including sodium glycocholate (GCA and GDCA), ethanol sodium deoxycholate (GCDCA), sodium taurodeoxycholate hydrate (TDCA), and sodium taurodeoxycholate (TCDCA and CA), are negatively correlated with *A. muciniphila* abundance and inhibit the growth of *A. muciniphila*. However, secondary bile DCA can increase the abundance of *A. muciniphila*. Ursodeoxycholic acid increases *A. muciniphila* abundance and improves colitis in mice [224]. A squalene synthase inhibitor (zaragozic acid A) increases the sensitivity of *A. muciniphila* to bile acid. Oleoylethanolamide (OEA), an endocannabinoid, is an important lipid signaling molecule in the gut–brain axis. Supplementation with OEA can increase *A. muciniphila* abundance and reduce energy and carbohydrate intake [225]. Physical exercise can regulate the gut flora in various diseases. Regular physical exercise increases intestinal Prevotella levels and decreases *A. muciniphila* abundance in patients with MS [226]. In the HF+DSS-induced mouse colitis model, a certain intensity of exercise training increases the diversity of gut flora (increases abundance of *A. muciniphila*) and reduces gut inflammation [227]. The gut flora may mediate the antidepressant effects of the C-terminal domain of the heavy chain of tetanus toxin (HC-TETX) [228].

## Conclusions and Perspectives

The gut is the primary site of microbial settlement and contains trillions of microbes. Bacteria, as the main inhabitants of the gut, interact with the host in many ways and play an important role in regulating body functions and defending against diseases. The gut flora is involved in human digestion and absorption, energy generation, vitamin synthesis, and immune regulation. It can even regulate the central nervous system and affect the physiological functions of the human brain. However, if the gut flora is out of balance, an increase or decrease in a certain type of bacteria can lead to disease. *A. muciniphila* also promotes intestinal stem cell proliferation and increases the number of goblet cells. *A. muciniphila* and its metabolites play an important role in endocrine system diseases, nervous system diseases, digestive system diseases, musculoskeletal system diseases, respiratory system diseases, and other diseases.

*A. muciniphila* abundance is decreased in most diseases but increased in PD, MS, necrotizing pancreatitis, and infantile eczema. The reasons for this difference need further experimental investigation. In original studies of *A. muciniphila* and disease, only complete live bacteria produced a probiotic effect; pretreated *A. muciniphila* did not. However, subsequent studies found that *A. muciniphila* used after pasteurization has higher activity than live *A. muciniphila* [73]. The difference may be due to the tolerance of *A. muciniphila* within a certain range of temperatures. Heat treatment at 121 °C and 225 kPa denatured the active components of *A. muciniphila*. The better effect of pasteurized *A. muciniphila* compared with live *A. muciniphila* may be due to some other components of the bacteria that interfere with Amuc_1100 but cannot tolerate pasteurization temperatures. P9 has potential for use in lipid-reducing and hypoglycemic drugs. Breakthroughs in determining the structure of P9 and the complex structure of P9 and ICAM-2 will greatly promote the clinical application of P9. *A. muciniphila* is expected to become the next generation of probiotics in addition to *Lactobacillus* and bifidobacteria. In 2019, the results from the first human trial of *A. muciniphila* probiotic supplements confirmed the safety of *A. muciniphila* administration [72]. This study brings hope for the clinical application of *A. muciniphila*, although it has some limitations, such as the small sample size and the lack of an accurate method to measure visceral and subcutaneous fat. Indirect regulation of *A. muciniphila* abundance may inhibit or even reverse disease progression. *A. muciniphila* abundance is regulated by dietary, drug, and probiotic factors. Although most of these regulators cannot specifically target *A. muciniphila* abundance, prior studies provide new ideas for us to target *A. muciniphila* regulation.
